# BET inhibition in advanced cutaneous T cell lymphoma is synergistically potentiated by BCL2 inhibition or HDAC inhibition

**DOI:** 10.18632/oncotarget.25670

**Published:** 2018-06-26

**Authors:** Sa Rang Kim, Julia M. Lewis, Benoit M. Cyrenne, Patrick F. Monico, Fatima N. Mirza, Kacie R. Carlson, Francine M. Foss, Michael Girardi

**Affiliations:** ^1^ Department of Dermatology, Yale School of Medicine, New Haven, CT 06510, USA; ^2^ Department of Internal Medicine, Section of Medical Oncology, Yale School of Medicine, New Haven, CT 06510, USA

**Keywords:** CTCL, apoptosis, BET inhibition, HDAC inhibition

## Abstract

While several systemic therapies are approved for cutaneous T cell lymphoma (CTCL), a non-Hodgkin lymphoma of skin-homing T cells that may involve lymph nodes and peripheral blood in advanced stages, relapses are common. Mutational analysis of CTCL cells has revealed frequent amplification of the *MYC* oncogene, and bromodomain and extraterminal (BET) protein inhibitors have been shown to repress MYC expression in various malignancies. Towards a potential novel therapy, we thus sought to examine the effect of BET inhibition on CTCL cells *in vitro*. Each of the four tested BET inhibitors (JQ1, ABBV-075, I-BET762, CPI-0610) consistently induced dose-dependent decreases in viability of isolated patient-derived CTCL cells and established CTCL cell lines (MyLa, Sez4, HH, Hut78). This effect was synergistically potentiated by combination of BET inhibition with BCL2 inhibition (e.g. venetoclax) or histone deacetylase (HDAC) inhibition (e.g. vorinostat or romidepsin). There was also a marked increase in caspase 3/7 activation when JQ1 was combined with either vorinostat or romidepsin, confirming that the observed synergies are due in major part to induction of apoptosis. Furthermore, *MYC* and *BCL2* expression were each synergistically repressed when CTCL cells were treated with JQ1 plus HDAC inhibitors, suggesting cooperative activities at the level of epigenetic regulation. Taken together, these data indicate that targeting BET proteins in CTCL represents a promising novel therapeutic strategy that may be substantially potentiated by combination with BCL2 or HDAC inhibition.

## INTRODUCTION

Cutaneous T cell lymphoma (CTCL), including the most common subtypes mycosis fungoides (MF) and Sézary syndrome (SS), represents a group of non-Hodgkin lymphomas of skin-homing, usually CD4+, malignant T cells [1, 2]. MF typically presents as cutaneous patches and plaques, but in more advanced disease, malignant T cells may disseminate to the blood, lymph nodes, and viscera [2, 3]. SS is a frank leukemic variant of CTCL that may progress from MF or develop *de novo* and is further characterized by erythroderma and bulky lymphadenopathy. Malignant T cells may comprise the majority of circulating T cells in patients with SS, with a median survival of 2 to 4 years [4–7]. The malignant T cells show constitutive activation and propensity for T-helper 2 cytokine production [8] that suppresses cell-mediated immunity and increases infection risk [1]. Unfortunately, CTCL remains generally incurable except in rare cases of allogeneic stem cell transplantation [9]. Overall response rates to single agent systemic therapies, including the retinoid bexarotene, and histone deacetylase (HDAC) inhibitors vorinostat and romidepsin, range between 20–45% and relapses are not uncommon [10, 11]. There is an unmet need for the treatment of advanced CTCL, and novel single or combination targeted therapies could be transformative.

Next-generation sequencing efforts have improved our understanding of the genetic alterations driving CTCL and may help shape novel approaches to therapeutic targeting of this malignancy [12–17]. CTCL is distinctive from the vast majority of other malignancies in that somatic copy number variants (SCNVs) comprise 92% of all driver mutations present within CTCL cells, and the resulting genetic derangements can be clustered into three pathways: T cell activation, cell cycle dysregulation/apoptosis, and DNA structural dysregulation affecting gene expression [12]. Within these pathways, prioritization of targeted therapies based on their specific mechanisms of action may be considered. Inhibition of the antiapoptotic protein B-cell lymphoma 2 (BCL2) was previously suggested as a targetable pathway based on common gene alterations that increase BCL2 activity and dependence, including *STAT3* and *STAT5B* amplification, *TP53* deletions and *CTLA4* deletions [18–22]. We recently showed that venetoclax (ABT-199), a BCL2-selective inhibitor approved for relapsed or refractory chronic lymphocytic leukemia (CLL) with 17p deletion, efficiently induces apoptosis in patient-derived CTCL cells *in vitro* and this effect is synergistically potentiated by combination with HDAC inhibition [23, 24].

Mutational analysis in CTCL has also revealed 12 significant broad SCNVs [12]. The most common of these are amplifications on chromosome 8q that include the *MYC* oncogene in 42.5% of leukemic CTCLs [12]. *MYC* family genes play critical roles in cell growth and survival, and therefore the frequent amplification of *MYC* in CTCL lends itself to therapeutic intervention [25]. Findings showing that NF-κB is a potent transcriptional activator of the *MYC* promoter [26] and that the NF-κB pathway is constitutively active in CTCL [27] further suggest *MYC* as a viable therapeutic target. Bromodomain and extra-terminal (BET) proteins are important in initiating and enhancing transcription and, in particular, the BET-protein BRD4 regulates key genes for cell cycle progression, including *MYC* [25, 28, 29]. JQ1, a small-molecule BET inhibitor, prevents BRD4 binding and shows potent antiproliferative effects via downregulation of *MYC* gene expression in several other hematologic and non-hematologic malignancies [30–35]. JQ1 has also been shown to have antiproliferative effects on CTCL cell lines [36]. However, the effects of BET inhibition on patient-derived CTCL cells or in combination with other targeted agents have not been reported previously.

Herein, we show that BET targeting substantially decreases the viability of advanced patient-derived CTCL cells *in vitro* and that this effect can be synergistically potentiated by either BCL2 inhibition or HDAC inhibition. The effect is consistent across a spectrum of BET inhibitors: all four BET inhibitors tested (JQ1, ABBV-075, I-BET762, CPI-0610) demonstrate activity against CTCL cells, with ABBV-075 being the most potent. Combination of BET inhibition and HDAC inhibition, in particular, showed significant attenuation of *MYC* and *BCL2* gene expression. Taken together, these data strongly suggest that BET inhibitors, alone and in combination with other agents, may allow for novel therapeutic strategies in the treatment of CTCL by cooperative repression of *MYC* and *BCL2* expression.

## RESULTS

### BET inhibition via JQ1 reduces viability of patient-derived CTCL cells and CTCL cell lines *in vitro*

**Figure 1:**
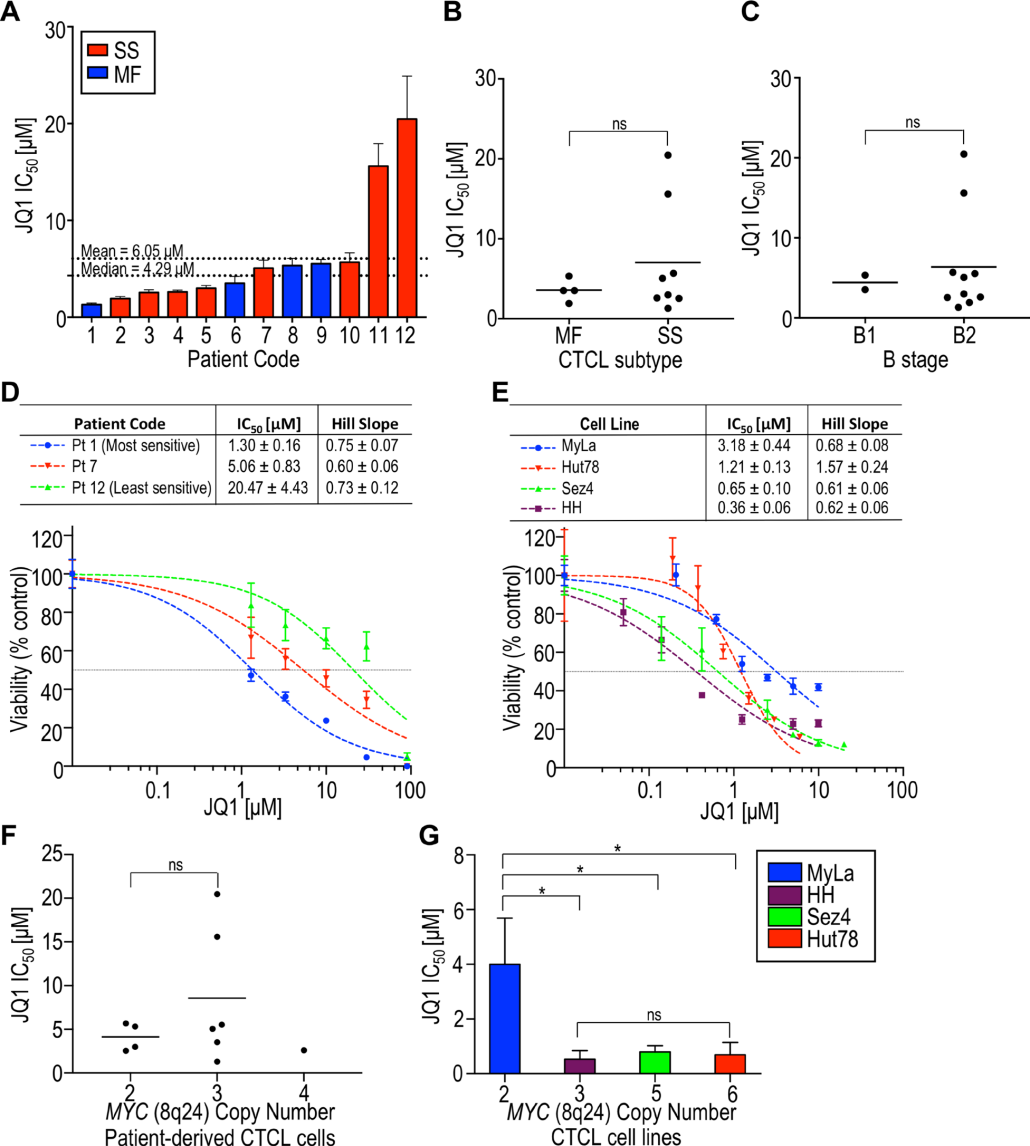
The BET inhibitor JQ1 substantially decreases the viability of patient-derived CTCL cells and CTCL cell lines All samples were incubated with JQ1 for 72 hours and dose-response curves were generated, from which IC_50_ and hill slopes were calculated. (**A**) IC_50_ of patient-derived samples in increasing order. The median and mean IC_50_s were 4.29 µM and 6.05 µM, respectively. Mycosis fungoides (MF) patients are in blue and Sézary syndrome (SS) patients are in red. (**B**) Comparison of IC_50_ with CTCL subtype at the time of diagnosis as either MF or SS. (**C**) Comparison of IC_50_ with CTCL B stage based on ISCL classification. (**D**) Representative dose-response curves for patient-derived samples. (**E**) Dose-response curves for cell lines. (**F**) Comparison of IC_50_ with *MYC* amplification status in patient-derived samples. (**G**) Comparison of IC_50_ with *MYC* amplification status in cell lines. ns, *p* > 0.05; pt, patient; ^*^p < 0.05.

To study the effects of BET inhibition, malignant cells were purified from the peripheral blood of 12 CTCL patients (Table 1) and exposed to JQ1 *in vitro*. We consistently observed a dose-dependent decrease in CTCL cell viability following a 72 hr exposure (Figure 1A). Patient samples showed varying sensitivities to JQ1, with IC_50_s ranging from 1.30 to 20.47 µM (mean 6.05 ± 5.88 µM). Patients were categorized according to their initial diagnosis as either MF or SS, and as either B1 or B2 using the 2007 International Society for Cutaneous Lymphomas (ISCL) classification and the 2016 Gibson *et al.* criteria [37, 38]. While the two highest IC_50_s were observed with malignant cells from patients with SS, we also observed five SS patient-derived samples with IC_50_s less than the mean. We found no correlation of IC_50_ with MF vs SS or B1 vs B2 status but there was notable heterogeneity with more advanced disease, which may reflect further acquisition of mutations and chromosomal abnormalities (Figure 1B, 1C) [39].

**Table 1:** Summary of CTCL patient characteristics

Pt ID	Sex	Age (yrs)	CTCL subtype at diagnosis	Stage at diagnosis	*MYC* copy number	TCR Vβ+	Current therapy	Previous therapy	CD4+/ CD8+ ratio	% Blood Involvement
**1**	M	78	MF	B2	3	Yes	Romidepsin, vorinostat	ECP, bexarotene, IFNγ-1b	23.97	25–30
**2**	F	78 (dc)	SS	B2	Unknown	Yes	Pralatrexate	Romidepsin, CHOEP, EPOCH, GND	60.20	26
**3**	M	67	SS	B2	2	Yes	ECP, nbUVB	nbUVB, topical steroids	3.89	17
**4**	F	65	SS	B2	4	Yes	ECP, bexarotene, intron-A	Topical steroids	1.27	39
**5**	M	73	SS	B2	2	Yes	Gemcitabine	ECP with bexarotene, romidepsin, pralatrexate	51.55	65
**6**	M	65	MF	B1	3	Yes	ECP, bexarotene, intron-A, IFNγ-1b	Topical steroids, nbUVB	3.17	11
**7**	M	85	SS	B2	3	Yes	Pralatrexate	PUVA, bexarotene, vorinostat, methotrexate, intron, ECP, romidepsin, doxorubicin, gemcitabine, alemtuzumab, brentuximab	322.00	53
**8**	F	63	MF	B1	2	Suspicion	ECP, topical nitrogen mustard, nbUVB, bexarotene	Topical steroids, topical nitrogen mustard	1.84	19–20
**9**	M	53	MF	B2	3	Yes	Pentostatin, cyclophosphamide	ECP, IFNγ-1b, bexarotene, romidepsin, pralatrexate, gemcitabine	161.33	75
**10**	F	72	SS	B2	2	Yes	Vorinostat	Romidepsin, belinostat, gemcitabine	21.65	31
**11**	F	62	SS	B2	3	Yes	ECP, bexarotene, intron-A	Phototherapy, oral and topical steroids	27.16	44–47
**12**	F	81	SS	B2	3	Yes	None	None	44.80	40

CTCL subtypes are subtypes at the time of diagnosis. B stage based on ISCL classification [37] and the 2016 criteria proposed by Gibson *et al.* [38]. TCR-Vβ**+** if >50% of the population of atypical cells express a single Vβ or if there is <20% expression of the entire 27 Vβ panel. Current therapy is defined as treatment at the time of experiment. CD4+/CD8+ ratio and % blood involvement of malignant cells are based on clinical flow cytometry of patients at the time of the experiment. CHOEP, cyclophosphamide, doxorubicin, etoposide, vincristine, prednisone; dc, deceased; ECP, extracorporeal photopheresis; EPOCH, etoposide, prednisone, vincristine, cyclophosphamide, doxorubicin; F, female; GND, gemcitabine, navelbine, doxorubicin; IFN, interferon; M, male; MF, mycosis fungoides; nbUVB, narrow band UV-B; SS, Sézary syndrome; Pt ID, patient identifier.

The effect of BET inhibition by JQ1 on cell viability was also studied in four established CTCL cell lines: MyLa 2059, HH, Sez4, and Hut78. Three cell lines (HH, Sez4, and Hut78), were considerably more sensitive to JQ1 than any of the patient-derived samples (Figure 1D, 1E). Since JQ1 is known to reduce *MYC* expression in other hematologic and solid malignancies, and gene amplification may augment gene expression, we determined *MYC* copy number by fluorescence *in situ* hybridization [40] and compared this to JQ1 sensitivity. While we found no correlation between JQ1 IC_50_ values and *MYC* amplification status in our patient-derived samples (Figure 1F), MyLa (normal *MYC* copy number) showed a significantly higher IC_50_ than each of the cell lines harboring *MYC* amplifications. However, a greater degree of *MYC* amplification (i.e. 6 vs 3 copies) did not render cells more sensitive to BET inhibition (Figure 1G). The CTCL subtype represented by each cell line may be relevant; MyLa originated from the skin of a patient with MF and HH from the blood of a patient with leukemic MF, while Sez4 and Hut78 were derived from patients with frank SS [41]. Of note, Hut78 showed a hillslope of >1, which may indicate allostery [42]. Taken together, the CTCL patient-derived and established cell-line datasets strongly suggest that *MYC* amplification status is not predictive of sensitivity to BET inhibition.

### A spectrum of BET inhibitors consistently reduces CTCL cell viability *in vitro*

**Figure 2:**
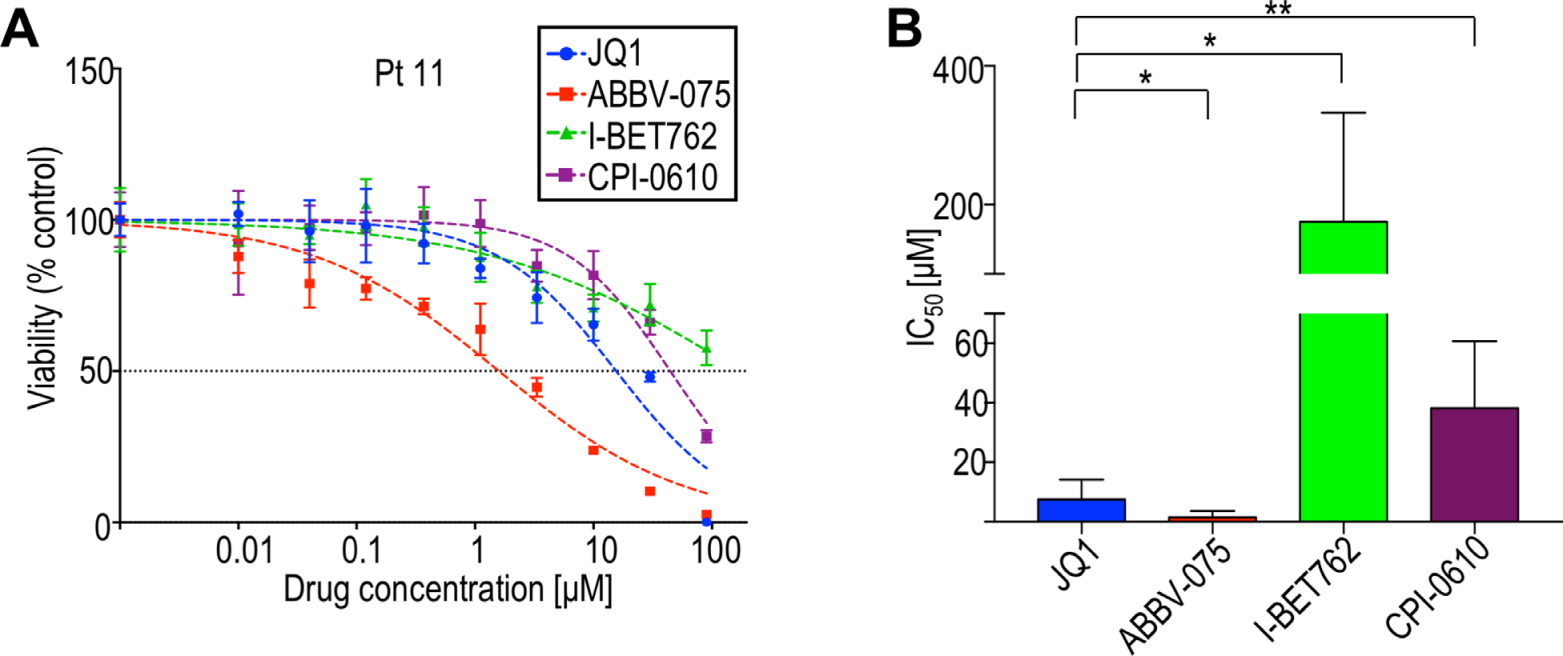
BET inhibitors in clinical development (ABBV-075, I-BET762, CPI-0610) are variably effective in limiting CTCL cell viability (**A**) Representative dose-response curves of CTCL cells derived from patient 11 to different BET inhibitors. (**B**) Comparison of average IC_50_s of BET inhibitors from patient-derived samples. ABBV-075 and JQ1 were tested against eight patient-derived samples; I-BET-762 and CPI-0610 were tested against five patient-derived samples. Patient codes and individual IC_50_s are provided in Supplementary Table 1. ^*^p < 0.05; ^**^p < 0.01.

BET inhibitors with more favorable pharmacological characteristics than JQ1 are being developed for clinical use, including I-BET762, CPI-0610, and ABBV-075 (in order of discovery) [43–45]. To evaluate relative activities of these BET inhibitors, we compared their effects on cell viability using eight CTCL patient-derived samples and four CTCL cell lines. In all samples tested, patient-derived and cell lines, ABBV-075 was the most potent on a per molar basis (Figure 2; Supplementary Table 1). Two-way ANOVA of dose-response curves for ABBV-075 and JQ1 showed a statistically significant difference in each patient-derived sample. I-BET762, which has a benzotriazoloazepine core similar to JQ1 [44], was the least potent against patient-derived samples. Comparison of the potency of ABBV-075 to other BET inhibitors has not been previously described, so it is yet unclear whether the observed differences may be generalized, or are specific to CTCL. Nonetheless, the consistent sensitivity of CTCL cells to this panel of BET inhibitors further implicates the BET pathway as a viable therapeutic target.

### Decreased CTCL cell viability by BET inhibition is synergistically potentiated by BCL2 or HDAC inhibition

**Figure 3:**
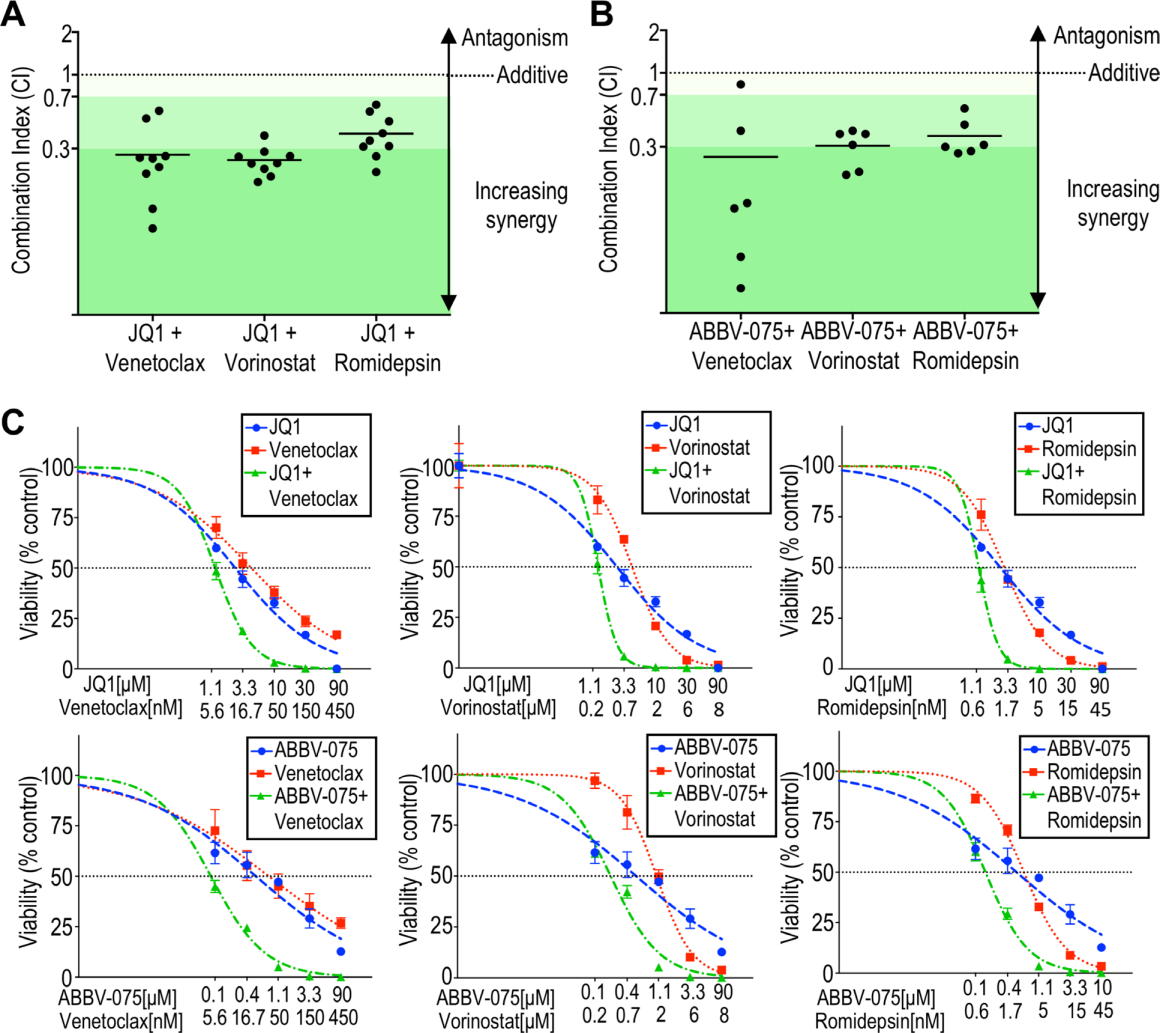
BCL2 inhibitors or HDAC inhibitors synergistically potentiate BET inhibition against patient-derived CTCL cells (**A**) The combination index (CI) at 10% viability was calculated for nine patient-derived samples (listed in Supplementary Table 2) exposed to JQ1 combined with venetoclax, vorinostat, or romidepsin, using the Chou-Talalay method. Strong synergy is defined as CI < 0.3, moderate synergy is CI < 0.7, and weak synergy is CI < 0.9 (adapted from Chou). (**B**) Calculated CI at 10% viability for six patient-derived samples exposed to ABBV-075 combined with venetoclax, vorinostat, or romidepsin. Synergy was note in all patient-derived samples. (**C**) Representative viability curves for BET inhibitors (JQ1 or ABBV-075) and their combinations with venetoclax, vorinostat, or romidepsin (patient 10).

We next sought to explore the potential potentiation of BET inhibition against CTCL. Malignant cells purified from nine CTCL patients were incubated with BET inhibitor JQ1 alone or in combination with the BCL2 inhibitor venetoclax, or one of two HDAC inhibitors, vorinostat or romidepsin, to assess for synergy by the cell viability assay, and hill slopes and IC_50_ values calculated. We observed a marked shift in the dose response curves when JQ1 was combined with a BCL2 inhibitor or HDAC inhibitor. The degree of synergy was quantified as combination index (CI) using the Chou-Talalay method, from dose-response curves with constant ratios of agents tested (CI = 1 indicates a purely additive effect, while CI < 1 reveals synergy) [46, 47]. All (9/9) patient-derived samples demonstrated synergy when JQ1 was combined with either a BCL2 inhibitor or HDAC inhibitor (Figure 3A, 3C; Supplementary Table 2) and the degree of synergy was either moderate (CI < 0.7) or strong (CI < 0.3). This result is striking given the heterogeneity of genetic aberrations in CTCL. Even in the case of patient 9, who was previously treated with romidepsin and relapsed, there still was moderate synergy (CI = 0.31). The degree of synergy did not correlate with sensitivity to JQ1 or *MYC* amplification status (data not shown).

We also assayed for synergy with ABBV-075, the most potent of the BET inhibitors assessed against CTCL cells *in vitro*. As for JQ1, synergy against CTCL was observed with ABBV-075 in combination with HDAC or BCL inhibition in the vast majority of patient-derived samples at the moderate or strong level (Figure 3B). Cell lines showed more varied results (Supplementary Table 2). While MyLa and Sez4 demonstrated synergy for all combinations, HH showed an antagonistic effect with JQ1 plus venetoclax, an additive effect with JQ1 plus vorinostat, and synergy with JQ1 plus romidepsin. Hut78 also showed an antagonistic effect with JQ1 plus venetoclax, and an additive effect when JQ1 was combined with either HDAC inhibitor. While cell lines serve as *in vitro* models for translational investigation and show similar gene expression patterns as advanced MF/SS patients, molecular changes inherent in cell culture may result in lines that do not reflect all aspects of freshly isolated samples [48]. It had been previously shown that JQ1 induces apoptosis in HH and Hut78, two cell lines that showed antagonism with JQ1 plus venetoclax, while JQ1 induces senescence in MyLa cells [36]. Despite that JQ1 and venetoclax each target components of the apoptosis pathway in HH and Hut78, these data suggest that for certain genetic or epigenetic alterations, this combination of agents may result in antagonism. Such antagonism was not observed in any of the CTCL patient samples tested.

### Combination BET inhibition and HDAC inhibition leads to marked increases in apoptosis induction

**Figure 4:**
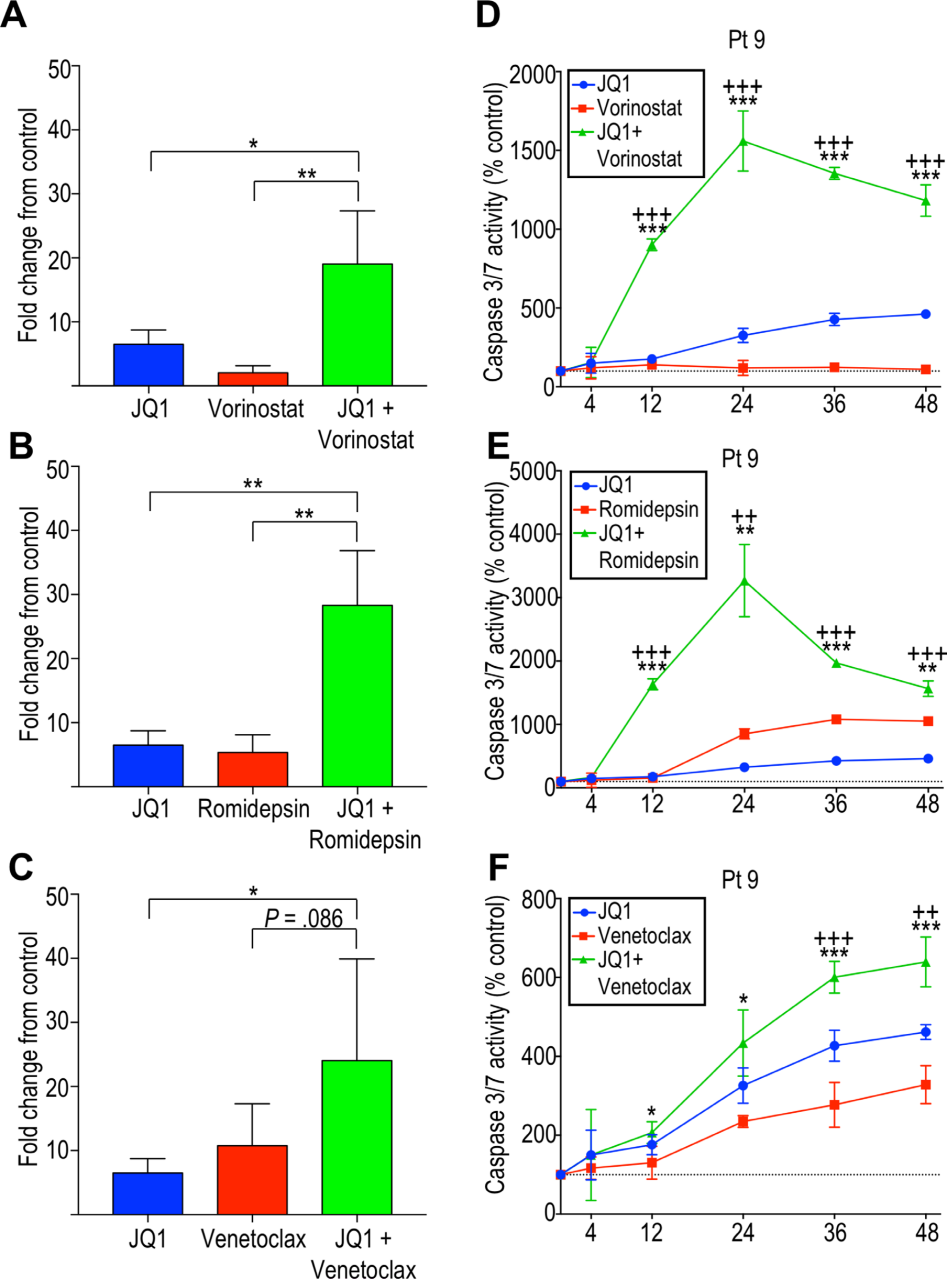
Effects of BET inhibition and its potentiation by HDAC inhibition are mediated in part by induction of apoptosis by caspase 3/7 Average caspase 3/7 activity at 24 hours, shown as fold change from the control, for four patient-derived samples (patient 4, 9, 11, and 12) incubated with (**A**) JQ1 and vorinostat, (**B**) JQ1 and romidepsin, and (**C**) JQ1 and venetoclax. Caspase 3/7 activity over 48 hours shown for patient 9 following incubation with (**D**) JQ1 and vorinostat, **(E)** JQ1 and romidepsin and (**F**) JQ1 and venetoclax. ^*^p < 0.05; ^**^p < 0.01; ^***^p < 0.001. For (D–F), +indicates *p*-value against JQ1 and ^*^indicates *p*-value against vorinostat, romidepsin or venetoclax.

To determine whether the observed dose-dependent decrease in cell viability was due in part to apoptosis induction, caspase 3/7 activation was measured. Patient-derived cells were incubated with single agents or combinations of agents, as described above. JQ1, venetoclax, and HDAC inhibitors independently induced caspase-dependent apoptosis (Figure 4). However, the combination of JQ1 with BCL2 inhibitor or HDAC inhibitors showed a higher rate of apoptosis than the rate observed with individual agents. In particular, JQ1 in combination with either HDAC inhibitor showed a striking increase in caspase-dependent apoptosis in all patient-derived samples tested (Figure 4A, 4B, 4D, 4E). While JQ1 plus venetoclax resulted in significantly increased caspase activity in some patients (Figure 4F), there was great variability and on average, this combination only approached statistical significance (Figure 4C). BET inhibition efficiently induces apoptosis in CTCL cells *in vitro*, and this is potentiated by HDAC inhibition, strongly suggesting this combinatorial therapy might benefit advanced CTCL patients, including those refractory to single agent therapy.

### *MYC* and *BCL2* gene expression are greatly attenuated by combination BET inhibition and HDAC inhibition

**Figure 5:**
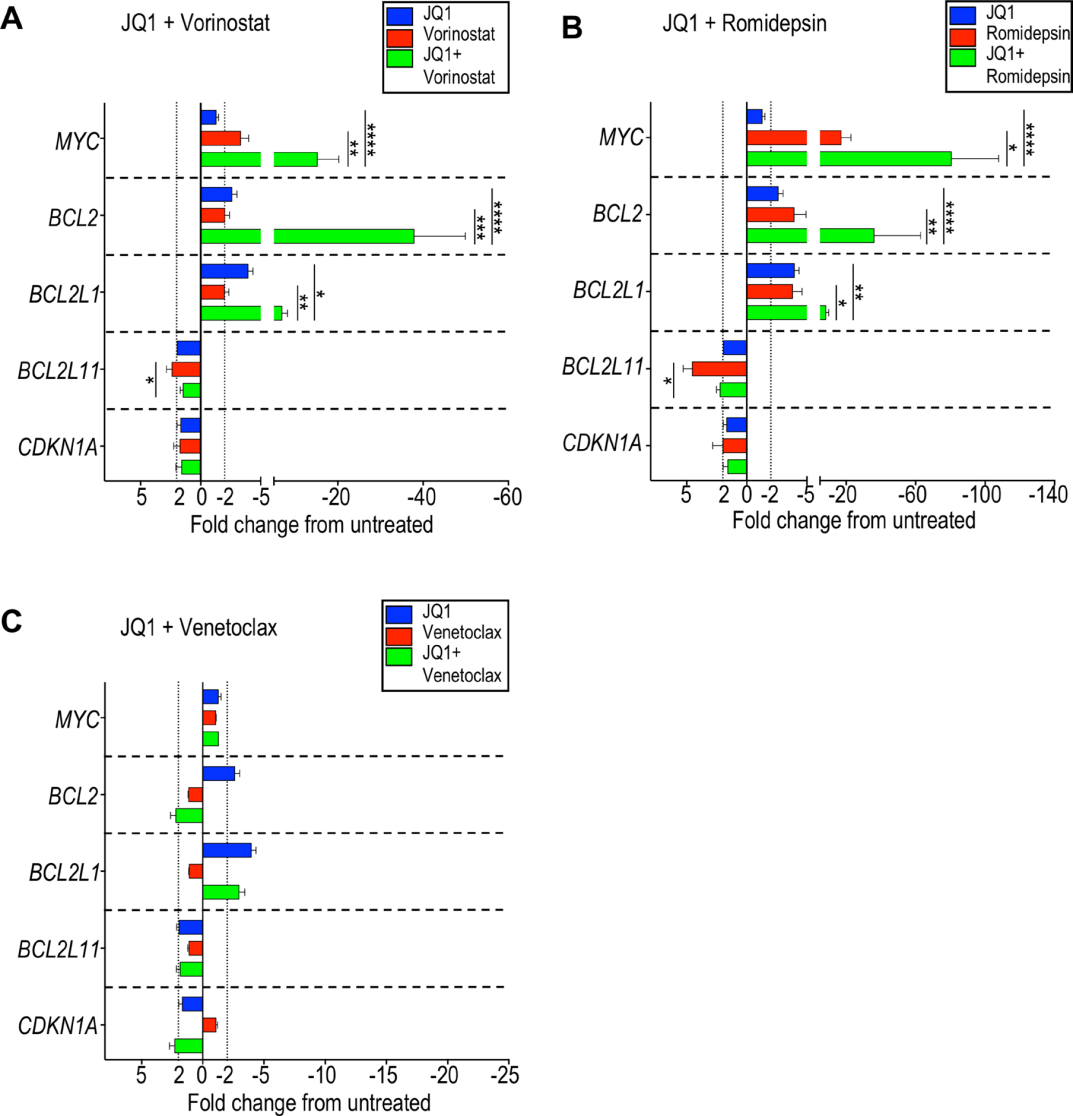
Combination of BET inhibition and HDAC inhibition markedly represses *MYC* and *BCL2* expression in CTCL cells Seven patient-derived samples (listed in Supplementary Table 3) were incubated with the indicated agents or vehicle control for 24 hours and changes in gene expression evaluated by qRT-PCR. Change in gene expression as represented by fold change from untreated for (**A**) JQ1 and vorinostat, (**B**) JQ1 and romidepsin, and (**C**) JQ1 and venetoclax. There was a striking decrease in *MYC* and *BCL2* expression when JQ1 was combined with either vorinostat or romidepsin. ^*^p < 0.05; ^**^p < 0.01; ^***^p < 0.001; ^****^p < 0.0001.

*MYC*, *BCL2*, *BCL2L1*, *BCL2L11*, and *CDKN1A* are genes that were previously reported to show altered expression under BET inhibition in several hematologic and solid cancer cell lines [32–34, 49–54]. In CTCL cell lines, specifically, MyLa, SeAx, Hut78, and HH, *MYC* expression was reported to decrease after exposure to JQ1 [36]. We were interested in examining changes in gene expression that may be important for the mechanisms of synergy we observed in patient-derived CTCL cells. Patient-derived CTCL samples were therefore incubated for 24 hours with 10 µM JQ1, 50 nM venetoclax, 2 µM vorinostat, or 5 nM romidepsin, as well as combinations of JQ1 with each of the other agents, and the relative expression of 5 genes were compared with a vehicle control. Notably, JQ1 alone did not affect *MYC* expression while vorinostat and romidepsin induced an average 3-fold and 17-fold decrease, respectively (Figure 5A, 5B; Supplementary Table 3). However, when JQ1 was combined with vorinostat, a 15-fold decrease in *MYC* expression was seen, and an 80-fold decrease was seen with JQ1 plus romidepsin, revealing synergistic repression of gene expression. This trend was also seen when ABBV-075 was combined with an HDAC inhibitor (Supplementary Table 4). This was also true for *BCL2* expression and, to a lesser degree, for *BCL2L1*. The *BCL2L11* gene encodes proapoptotic BIM, which binds to BCL2 and is suggested to play a key role in the mechanism of synergy of BET inhibition and BCL2 inhibition. While we did observe an increase in *BCL2L11* expression following HDAC inhibition, we did not see significant changes following treatment with JQ1, except in two patient samples. As expected, no significant changes in expression of *BCL2*, *BCL2L1*, and *BCL2L11* were observed with venetoclax (Figure 5C), consistent with its known mechanism of action of inhibiting BCL2 protein binding, thereby sequestering proapoptotic proteins BAX or BAK [55].

## DISCUSSION

The presented pre-clinical data provides substantial evidence for the potential of BET inhibitors in the treatment of advanced CTCL. BET protein BRD4 regulates transcription of key genes for cell cycle progression, such as the *MYC* oncogene that is often amplified in CTCL, by recruiting the positive transcription elongation factor and phosphorylating the C terminal domain serine 2 on RNA polymerase II [25, 28, 29]. Anti-tumor activity and repression of *MYC* by BET inhibitors have been shown in various malignancies including multiple myeloma (MM), Burkitt’s lymphoma, and acute myelogenous leukemia (AML) [32, 33, 56, 57]. Decreased *MYC* expression occurs due to BRD4 depletion in enhancer regions that drive *MYC* expression [57, 58]. This effect may be intensified in *MYC*-amplified tumors; in *MYC*-amplified medulloblastoma cell lines, JQ1 had a greater effect on limiting cell proliferation [59].

Combination approaches using BET inhibitors and other targeted therapies also have been described in multiple hematologic and solid tumors, but not previously in CTCL [49–52, 60]. For example, synergy between JQ1 and the BCL2 inhibitor navitoclax (ABT-263) against *MYCN*-amplified SCLC has been reported [49] and preclinical studies combining BET inhibitors and HDAC inhibitors showed synergistic activity against urothelial carcinoma cell lines, melanoma cells, and murine models of pancreatic ductal adenocarcinoma [50, 51, 60]. Based on genetic alterations in CTCL that may affect BCL2, we identified BCL2 as promising target in CTCL, and revealed that the BCL2 inhibitor venetoclax exhibits marked activity against CTCL viability [24], an effect synergistically potentiated by HDAC inhibitors, vorinostat and romidepsin (both agents have been previously approved by the U.S. FDA for CTCL) [10]. In the current studies, we show that the cytotoxic effect of BET inhibition in CTCL cells is synergistically potentiated by either BCL2 inhibition or HDAC inhibition in the vast majority of both patient-derived samples and CTCL cell lines. No correlation was observed between *MYC* copy number and IC_50_ in our CTCL patient-isolated cells; *MYC* amplification may not necessarily translate to increased *MYC* expression. However, even samples derived from patients who have tried and failed multiple single therapies showed a marked decrease in *MYC* expression when exposed to drug combinations. One prior case-based study reported 2 patients with NUT midline carcinoma treated with OTX015, a BET inhibitor, with rapid clinical response in less than 2 weeks. However, both later experienced disease progression and biopsies revealed high MYC levels, suggesting resistance to MYC suppression [61]. Combination therapy may be a promising approach to overcome resistance in such cases.

To study more clinically applicable BET inhibitors in CTCL, we selected and assessed those currently in clinical trials for other cancers. I-BET762 is undergoing phase I/II studies for cancers including relapsed refractory AML and MM as well as ER+ breast cancer and prostate cancer (NCT01943851, NCT02964507, NCT03150056). A phase I study using CPI-0610 in diffuse large B-cell lymphoma and follicular lymphoma, found doses of 170 mg and 230 mg once daily were associated with plasma concentrations of ≥3 µM and were generally well tolerated [62]. ABBV-075 is undergoing phase I study to evaluate the safety profile in cancers including AML, prostate cancer, and SCLC (NCT02391480). While I-BET762 has a very similar core structure to JQ1 [44], it was not as potent in CTCL patient-derived cells and cell lines in this study. CPI-0610 has a 3,4-dimethylisozazole moiety added to its core, allowing for additional hydrogen bonding, but it was not more potent than JQ1 [45]. Multiple BRD4 binding sites may allow for more potency and selectivity. ABBV-075 has a more distinct pyrrolopyridone core that additionally binds the conserved Asn433 residue of BET proteins [43, 63], which may account for the higher potency seen in this study.

We found that ABBV-075 also shows synergy with BCL2- or HDAC- inhibition, as for JQ1. This further supports the hypothesis that synergy observed with BET plus BCL2- or HDAC- inhibition is due to targeting of specific pathways that are affected by BRD4. In our study, we observed synergistic effects on expression of *MYC* and *BCL2*, and to a lesser degree *BCL2L1*, following exposure to combined BET inhibitors and HDAC inhibitors. A prior study of gene expression in lymphoma cells indicated a ∼25% overlap of genes induced by either BET inhibitors or HDAC inhibitors and suggested that the mechanism of synergy of BET plus HDAC inhibition in MYC-overexpressing cells is partly due to induction of HDAC-silenced genes [64]. While differential effects on gene expression may be responsible for synergy, BET and HDAC inhibition may also work in concert through BRD4. BRD4 binds to target gene promoters or super-enhancers, including those of oncogenes such as *MYC* and *BCL2* [57, 58, 65]. BET inhibitors prevent BRD4 from binding to acetyl-lysine and recruiting transcriptional machinery by occupying the binding pocket. In fact, treatment with JQ1 preferentially reduced BRD4 at super-enhancers for *MYC* in MM cells [57] and reduced BRD4 occupancy at promoters of *MYC*, *BCL2*, and *CDK6* in AML cell lines [66] while HDAC inhibitors caused a substantial increase in global acetylation of genes, resulting in translocation and redistribution of BRD4 as it binds to newly acetylated sites [67]. In another study, while there was an overall increase in marks bound by BRD4 following treatment with HDAC inhibitors, there was a loss of proper localization of BRD4 for specific gene transcription [68]. Direct blocking of BRD4 binding by BET inhibitors, as previously described [69, 70], and translocation of BRD4 due to global acetylation by HDAC inhibitors are two independent mechanisms that converge on BRD4 and may be responsible for the marked attenuation of *MYC* and *BCL2* transcription observed in CTCL cells.

Other genes of interest included *BCL2L11*, encoding proapoptotic BIM, and *CDKN1A*, encoding cell cycle regulator p21. BIM expression has been shown to be upregulated 2-fold in *MYC*-amplified SCLC following treatment with ABBV-075 [71]. Other BET inhibitors have been shown to upregulate BIM in AML cells and melanoma [51, 66]. BIM has been suggested to play a key role in the mechanism of synergy between BET inhibition and BCL2 inhibition in primary double-hit lymphoma cells, and SCLC [71, 72]. BIM binds BCL2, altering the balance between pro-apoptotic and anti-apoptotic signals. Although there was an increase in BIM expression, it was on average less than 2-fold in our patient-derived CTCL cells following culture with JQ1. Nonetheless, synergy was observed with JQ1 and venetoclax. HDAC inhibition has previously been shown to upregulate BIM [73]. In all patient samples, while HDAC inhibition led to increased *BCL2L11* expression, this was diminished when combined with JQ1. This suggests that while there is an antagonistic effect on *BCL2L11* expression, the net decrease in expression of *MYC* and *BCL2* predominates and cooperates, leading to the observed synergistic effects.

In summary, BET inhibition effectively limits the viability of leukemic CTCL cells, in part via induction of apoptosis. There was a clear synergistic effect when a BET inhibitor was combined with either a BCL2 inhibitor or an HDAC inhibitor, and expression data further suggested synergy at the epigenetic level with HDAC inhibitors. Our pre-clinical data strongly suggests that therapeutic targeting of CTCL using BET inhibition, alone or in combination with BCL2 inhibition or HDAC inhibition, represents a promising strategy in the treatment of CTCL that warrants clinical testing.

## MATERIALS AND METHODS

### CTCL patient samples

Peripheral blood was obtained from CTCL patients at the Yale Cancer Center. All procedures were approved by the Yale Human Investigational Review Board, and informed consent was obtained. Blood was collected in lithium heparin tubes and peripheral blood mononuclear cells (PBMC) were isolated by Ficoll density gradient. Malignant T cells were purified with a CD4+ negative selection kit (Miltenyi Biotec, Bergisch Gladbach, Germany) supplemented with antibodies to remove CD26+ and/or CD7+ cells, depending on the known malignant cell phenotype. Purity was assessed by flow cytometry using phenotypic markers of individual patient’s malignant cells (previously clinically identified), including Vβ in 10 of 12 patients, and was consistently >97%. For assays, cells were cultured in RPMI 1640 containing 10% heat-inactivated FBS (HI-FBS), 2 mM L-glutamine, 100 U/mL penicillin, 100 mg/mL streptomycin (L-glutamine/Pen/Strep), and the following interleukins (IL): IL2 (10 ng/mL), IL7 (5 ng/mL), IL15 (10 ng/mL), and IL13 (10 ng/mL; all from R&D Systems, Minneapolis) at 37° C, 5% CO_2_, and 95% humidity. Clinical fluorescence *in situ* hybridization (FISH) testing was used to determine the patients’ *MYC* copy number status.

### CTCL cell lines

MyLa, HH, Hut78, and Sez4 CTCL cell lines were described previously [74–78]. MyLa (Myla2059) was provided by Dr. E. Contassot (University Hospital, Zurich, Switzerland), HH and Hut 78 were purchased from American Type Culture Collection and Sez4 was provided by Dr. A. Rook (University of Pennsylvania, Philadelphia, PA). We have previously characterized genetic alterations in HH, Hut78, and Sez4 [78]. MyLa and HH were cultured in RPMI 1640 plus 10% HI-FBS and L-glutamine/Pen/Strep. Sez4 used the same medium supplemented with IL2 (20 ng/mL). Hut78 required Iscove’s Modified Dulbecco’s Medium plus 20% HI-FBS and L-glutamine/Penicillin/Strep. All cells were cultured at 37° C, 5% CO_2_, and 95% humidity. All media and supplements were obtained from Invitrogen (Carlsbad, CA). FISH testing to determine *MYC* copy number status was performed by the Molecular Cytogenetics Laboratory at Yale University School of Medicine.

### Flow cytometry

Unfractionated PBMC and purified malignant T-cells were analyzed by flow cytometry using the Stratedigm-13 (Stratedigm Inc, San Jose CA). Cells were blocked with human TruStain FcX (BioLegend, San Jose, CA) for 10 minutes, incubated with monoclonal antibodies directed against CD3 (BD Biosciences, San Jose, CA), CD4, CD7, CD26 (eBioscience, San Jose, CA), TCR-Vβ (Beckman Coulter, Brea, CA) or matched isotype controls for 20 minutes at 4° C, washed three times and fixed in 1% paraformaldehyde. FlowJo Software (v10; FlowJo, LLC) was used for data analysis.

### Cell viability assay

Cells were plated at 10,000/well in 96-well black optical plates and cultured for 72 hours in vehicle control (0.2% dimethyl sulfoxide (DMSO) or the following range of drug concentrations, alone or in combination: 0.01 to 90 µM BET inhibitor (JQ1, ABBV-075, I-BET762, CPI-0610), 5.6 to 450 nm venetoclax, 0.2 to 18 µM vorinostat, and 0.6 to 45 nM romidepsin. All drugs were obtained from ApexBio (Houston, TX) except ABBV-075 (Cayman Chemicals, Ann Arbor, MI). Drug concentrations were applied in approximate half-log10 increments to patient samples and two-fold increments for cell lines. Viability was measured using the CellTiter-Glo Luminescent Cell Viability Assay (Promega, WI) as per the manufacturer’s protocol and plates were read with the Victor X Light Luminescence Counter (Perkin Elmer, Waltham, MA). Cell luminescence was normalized to vehicle control and corrected for media.

### Apoptosis assay

Patient derived cells were incubated for 24 hr as described for the cell viability assay. Following incubation, the Promega Caspase-Glo 3/7 assay (Madison, WI) was used to quantitate caspase activity, as per the manufacturer’s protocol. Plates were read using the Victor X Light Luminescence Counter.

### Gene expression profiling

RNA was isolated using the Qiagen RNeasy Micro (patient-derived cells) or Mini kit (cell lines), per the manufacturer’s protocol (Hilden, Germany). The High Capacity cDNA Reverse Transcription Kit and TaqMan PreAmp Master Mix (Applied Biosystems Inc., Foster City, CA) were used for cDNA synthesis and preamplification, respectively. Quantitative real time PCR (qRT-PCR) was performed (ABI 7500, SDS 2.0 software) using TaqMan Gene Expression Master Mix and TaqMan primers. Hypoxanthine-guanine phosphoribosyltransferase 1 (*HPRT1*) was used as the reference gene to normalize cycle threshold (Ct) values and expression differences relative to controls calculated using RQ = 2^−ΔΔCt^. Statistical analysis was done using RQ values.

### Calculation of IC_50_ values and drug synergy

For the cell viability and apoptosis assays, each drug concentration was performed in quadruplicate, and the mean values were plotted with their respective standard deviation. The mean inhibitor concentration (IC_50_) was determined using GraphPad Prism (version 7.01). Drug combinations were done in fixed dose ratios, which were determined based on the IC_50_ of the individual drugs. At least five different concentrations were performed and combination index (CI) values were calculated using the Chou-Talalay method in Microsoft Excel [46, 47].

### Statistical analysis

Graphpad Prism (version 7.01) was used for all statistical calculations. *P* values were calculated by parametric, unpaired two-tailed *t* tests.

## SUPPLEMENTARY MATERIALS TABLES



## Abbreviations

AML: acute myelogenous leukemia
BCL2: B-cell lymphoma 2
BET: bromodomain and extraterminal proteins
CTCL: cutaneous T cell lymphoma
HDAC: histone deacetylase
MF: mycosis fungoides
MM: multiple myeloma
SCLC: small cell lung cancer
SS: Sézary syndrome
