# Supplementary material for: BET inhibition in advanced cutaneous T cell lymphoma is synergistically potentiated by BCL2 inhibition or HDAC inhibition
Source: Oncotarget. 2018 Jun 26;9(49):29193–207. doi: 10.18632/oncotarget.25670 (PMC6044378; doi:10.18632/oncotarget.25670)
Supplement: Supplementary file 1 [file oncotarget-09-29193-s001.pdf]

BET inhibition in advanced cutaneous T cell lymphoma is synergistically potentiated by BCL2 inhibition or HDAC inhibition

SUPPLEMENTARY MATERIALS

SUPPLEMENTARY REFERENCES

1. Chou TC. Drug combination studies and their synergy quantification using the Chou-Talalay method. Cancer Res. 2010; 70:440–446.

2. Chou TC. Theoretical basis, experimental design, and computerized simulation of synergism and antagonism in drug combination studies. Pharmacol Rev. 2006; 58:621–681.

Supplementary Table 1: Mean IC_{50} ± SEM [µM], calculated from dose-response curves for each BET inhibitor (JQ1, ABBV-075, I-BET762, CPI-0610)

	JQ1	ABBV-075	I-BET762	CPI-0610
**Patient-derived cells**				
Pt 1	2.35 ± 0.36	0.47 ± 0.07		
Pt 4	2.63 ± 0.17	0.13 ± 0.01		
Pt 5	3.12 ± 0.34	0.55 ± 0.06		
Pt 7	4.76 ± 0.74	1.00 ± 0.15	95.15 ± 21.66	37.56 ± 8.74
Pt 9	5.45 ± 0.55	0.68 ± 0.08	55.33 ± 6.68	21.77 ± 3.01
Pt 10	5.49 ± 0.88	0.41 ± 0.06	106.1 ± 36.80	14.66 ± 3.55
Pt 11	15.56 ± 2.18	1.59 ± 0.21	174.3 ± 65.24	44.54 ± 5.57
Pt 12	20.31 ± 4.03	6.74 ±1.06	446.7 ± 366.1	72.33 ±17.3
**CTCL cell lines**				
MyLa	4.12 ± 0.61	0.29 ± 0.02	11.1 ±1.78	17.55 ±2.36
HH	0.89 ± 0.15	0.01 ± 0.004	0.59 ± 0.10	1.62 ± 0.34
Sez4	0.81 ± 0.15	0.05 ± 0.01	3.73 ± 0.45	6.25 ± 0.79
Hut78	1.21 ± 0.13	0.02 ± 0.003	0.77 ± 0.15	1.13 ± 0.22

ABBV-075 has the lowest IC_{50}, followed by JQ1 in all patient-derived samples. Pt, patient.

Supplementary Table 2: Combination index (CI) ± SEM as calculated by Chou-Talalay method at 10% viability (0.9 fraction affected) for patient-derived cells and CTCL cell lines

	JQ1 + Venetoclax	ABBV-075 + Venetoclax
**Patient-derived cells**		
Pt 1	0.22 ± 0.04	0.11 ± 0.01
Pt 3	0.26 ± 0.02	
Pt 4	0.26 ± 0.02	
Pt 5	0.56 ± 0.05	0.83 ± 0.11
Pt 7	0.27 ± 0.04	
Pt 9	0.49 ± 0.08	0.39 ± 0.06
Pt 10	0.11 ± 0.01	0.03 ± 0.01
Pt 11	0.20 ± 0.02	0.12 ± 0.01
Pt 12	0.08 ± 0.01	0.05 ±0.01
**Cell Lines**		
MyLa	0.72 ± 0.35	
HH	1.57 ± 0.20	
Sez4	0.56 ± 0.12	
Hut78	1.30 ± 0.42	

Strong synergy	<0.3
Moderate synergy	0.3–0.7
Weak synergy	0.7–0.9
Additive	0.9–1.1
Weak antagonism	1.1–1.45
Moderate antagonism	1.45–3.3
Strong antagonism	>3.3

	JQ1 + Vorinostat	ABBV-075 + Vorinostat
**Patient-derived cells**		
Pt 1	0.24± 0.03	0.19 ± 0.03
Pt 3	0.29 ± 0.04	
Pt 4	0.22 ± 0.02	
Pt 5	0.37 ± 0.17	0.37 ± 0.29
Pt 7	0.19 ± 0.01	
Pt 9	0.24 ± 0.03	0.20 ± 0.03
Pt 10	0.17 ± 0.02	0.37 ± 0.04
Pt 11	0.26 ± 0.02	0.39 ± 0.03
Pt 12	0.27 ± 0.03	0.31 ± 0.05
**Cell Lines**		
MyLa	0.28 ± 0.07	
HH	1.06 ± 1.24	
Sez4	0.34 ± 0.12	
Hut78	0.90 ± 1.18	

	JQ1 + Romidepsin	ABBV-075 + Romidepsin
**Patient-derived cells**		
Pt 1	0.26± 0.03	0.27 ± 0.03
Pt 3	0.61 ± 0.68	
Pt 4	0.39 ± 0.08	
Pt 5	0.55 ± 0.29	0.56 ± 0.36
Pt 7	0.31 ± 0.06	
Pt 9	0.31 ± 0.09	0.31 ± 0.11
Pt 10	0.21 ± 0.02	0.28 ± 0.02
Pt 11	0.47 ± 0.10	0.43 ± 0.08
Pt 12	0.34 ± 0.09	0.30 ± 0.08
**Cell Lines**		
MyLa	0.39 ± 0.13	
HH	0.25 ± 1.93	
Sez4	0.46 ± 0.56	
Hut78	0.99 ± 0.86	

As per legend, strong synergy CI < 0.3, moderate synergy 0.3–0.7, weak synergy 0.7–0.9, additive effect 0.9–1.1, weak antagonism 1.1–1.45, moderate antagonism 1.45–3.3, and strong antagonism >3.3 (adapted from Chou) [1, 2]. All patient-derived samples demonstrated synergy when JQ1 was combined with venetoclax, vorinostat, or romidepsin.

Supplementary Table 3: Change in expression of *MYC*, *BCL2*, *BCL2L1*, *BCL2L11*, *CDKN1A* shown as fold change from untreated, following 24 hour treatments with 10 µM JQ1, 50 nM venetoclax, 2 µM vorinostat and 5 nM romidpesin as single drugs or in combinations

	JQ1	Venetoclax	JQ1+ Venetoclax	Vorinostat	JQ1+ Vorinostat	Romidepsin	JQ1+ Romidepsin
***Gene* (protein):**							
***MYC* (MYC)**							
Pt 1	1.48	1.12	1.36	−2.19	−16.51	−6.32	−55.12
Pt 2	−1.90	−1.06	−1.89	−3.83	−21.38	−28.81	−29.48
Pt 4	−1.37	−1.06	−1.46	−2.17	−6.06	−52.08	−158.37
Pt 7	−2.64	−1.33	−2.40	−8.15	−29.37	−24.16	−149.73
Pt 9	−1.18	1.00	1.08	−2.09	−8.02	−10.40	−67.18
Pt 11	−1.16	−1.04	−1.35	−5.84	−61.41	−60.08	−296.34
Pt 12	−1.58	−1.09	−1.65	−6.50	−75.66	−23.46	−282.13
Avg*	**−1.28**	**−1.05**	**−1.29**	**−3.33**	**−15.19**	**−17.12**	**−80.59**
***BCL2* (BCL2)**							
Pt 1	−1.48	1.29	−1.08	−1.63	−31.76	−2.66	−64.58
Pt 2	−2.28	−1.04	−2.84	−1.36	−31.54	−2.70	−6.62
Pt 4	−4.10	1.12	−4.23	−2.56	−14.34	−13.96	−108.21
Pt 7	−4.53	1.05	−5.29	−6.10	−165.88	−12.40	−317.25
Pt 9	−3.15	1.17	1.08	−1.13	−40.68	−2.02	−128.99
Pt 11	−2.16	1.35	−1.87	−2.36	−55.84	−10.42	−188.14
Pt 12	−3.11	1.15	−2.60	−3.62	−301.53	−3.41	−502.04
Avg*	**−2.61**	**1.16**	**−2.19**	**−2.01**	**−37.88**	**−3.93**	**−36.06**
***BCL2L1* (BCL-XL)**							
Pt 1	−3.61	1.13	−2.90	−2.28	−6.78	−3.76	−8.94
Pt 2	−3.62	−1.14	−2.38	−1.81	−3.26	−2.93	−3.65
Pt 4	−4.73	−1.02	−4.82	−2.11	−8.07	−8.85	−19.66
Pt 7	−4.38	1.14	−2.72	−5.02	−10.58	−8.06	−12.61
Pt 9	−2.59	1.34	−1.66	1.04	−6.62	−2.02	−7.31
Pt 11	−5.67	1.18	−5.49	−2.37	−10.79	−6.88	−9.47
Pt 12	−4.57	1.03	−4.03	−2.33	−9.68	−2.80	−10.39
Avg*	**−3.95**	**1.10**	**−2.95**	**−1.97**	**−6.87**	**−3.80**	**−8.19**
***BCL2L11* (BIM)**							
Pt 1	3.01	1.28	3.45	4.68	1.72	6.232	2.37
Pt 2	1.92	1.69	1.14	1.59	−1.67	6.12	1.87
Pt 4	1.69	−1.19	1.47	1.46	1.01	1.42	1.25
Pt 7	1.68	1.01	1.75	2.15	1.76	3.66	2.18
Pt 9	1.78	1.08	1.83	1.55	1.07	4.57	2.47
Pt 11	1.45	−1.01	1.39	1.86	1.39	2.78	1.58
Pt 12	2.04	−1.01	1.99	3.39	2.61	6.84	3.80
Avg*	**1.94**	**1.12**	**1.86**	**2.38**	**1.45**	**4.53**	**2.22**
***CDKN1A* (P21)**							
Pt 1	1.20	1.12	1.88	−1.18	−2.04	−1.26	−3.33
Pt 2	2.35	1.19	4.01	1.16	−1.08	1.27	1.95
Pt 4	1.83	−1.13	1.69	2.31	3.31	1.46	1.83
Pt 7	−1.12	−1.49	1.47	−1.31	−1.63	1.20	−1.46
Pt 9	−1.99	1.21	−1.24	−1.04	1.29	1.25	2.06
Pt 11	2.05	−3.78	2.60	1.31	−1.14	−1.10	−1.38
Pt 12	2.81	1.20	3.53	4.67	3.44	7.02	3.47
Avg*	**1.66**	**−1.07**	**2.28**	**1.72**	**1.57**	**1.99**	**1.57**

Pt, patient.

*average fold changes were calculated from average RQ values.

Supplementary Table 4: Change in expression of *MYC* and *BCL2* shown as fold change from untreated, following 24 hour treatments with 2.5 µM ABBV-075, 50 nM venetoclax, 2 µM vorinostat and 5 nM romidpesin as single drugs or in combinations

	**ABBV−075**	**Venetoclax**	**ABBV−075+ Venetoclax**	**Vorinostat**	**ABBV−075+ Vorinostat**	**Romidepsin**	**ABBV−075+ Romidepsin**
***Gene* (protein):** ***MYC* (MYC)**							
Pt 1	1.26	−1.09	1.00	−2.20	−5.61	−4.44	−6.19
Pt 11	−1.43	1.01	−1.30	−5.12	−25.23	−33.89	−75.18
Pt 12	−2.95	1.24	−3.02	−3.16	−13.71	−12.00	−48.95
Avg*	**−1.03**	**−1.06**	**−1.49**	**−3.11**	**−10.31**	**−8.87**	**−15.37**
***BCL2* (BCL2)**							
Pt 1	−2.03	1.11	−2.13	−1.90	−17.15	−1.96	−9.46
Pt 11	−4.50	1.24	−4.29	−3.04	−20.33	−4.71	−29.99
Pt 12	−8.92	1.07	−7.55	−2.94	−52.61	−2.89	−68.75
Avg*	**−3.91**	**1.14**	**−3.60**	**−2.51**	**−23.72**	**−2.81**	**−19.53**

Pt, patient. *average fold changes were calculated from average RQ values.
